# Dynamic network model reveals distinct tau spreading patterns in early- and late-onset Alzheimer disease

**DOI:** 10.1186/s13195-022-01061-0

**Published:** 2022-09-02

**Authors:** Wha Jin Lee, Hanna Cho, Min Seok Baek, Han-Kyeol Kim, Jae Hoon Lee, Young Hoon Ryu, Chul Hyoung Lyoo, Joon-Kyung Seong

**Affiliations:** 1 School of Biomedical Engineering, Korea University, 145 Anam-ro, Seongbuk-gu, Seoul, South Korea; 2 Department of Neurology, Gangnam Severance Hospital, Yonsei University College of Medicine, 20 Eonjuro 63-gil, Gangnam-gu, Seoul, South Korea; 3 Department of Neurology, Wonju Severance Christian Hospital, Yonsei University Wonju College of Medicine, Wonju, Gangwon-do South Korea; 4 Department of Nuclear Medicine, Gangnam Severance Hospital, Yonsei University College of Medicine, Seoul, South Korea; 5 Department of Artificial Intelligence, Korea University, 145 Anam-ro, Seongbuk-gu, Seoul, South Korea; 6 Interdisciplinary Program in Precision Public Health, Korea University, Seoul, South Korea

**Keywords:** Alzheimer’s disease, Tau, Amyloid, Positron emission tomography, Network community

## Abstract

**Background:**

The clinical features of Alzheimer’s disease (AD) vary substantially depending on whether the onset of cognitive deficits is early or late. The amount and distribution patterns of tau pathology are thought to play a key role in the clinical characteristics of AD, which spreads throughout the large-scale brain network. Here, we describe the differences between tau-spreading processes in early- and late-onset symptomatic individuals on the AD spectrum.

**Methods:**

We divided 74 cognitively unimpaired (CU) and 68 cognitively impaired (CI) patients receiving ^18^F-flortaucipir positron emission tomography scans into two groups by age and age at onset. Members of each group were arranged in a pseudo-longitudinal order based on baseline tau pathology severity, and potential interregional tau-spreading pathways were defined following the order using longitudinal tau uptake. We detected a multilayer community structure through consecutive tau-spreading networks to identify spatio-temporal changes in the propagation hubs.

**Results:**

In each group, ordered tau-spreading networks revealed the stage-dependent dynamics of tau propagation, supporting distinct tau accumulation patterns. In the young CU/early-onset CI group, tau appears to spread through a combination of three independent communities with partially overlapped territories, whose specific driving regions were the basal temporal regions, left medial and lateral temporal regions, and left parietal regions. For the old CU/late-onset CI group, however, continuation of major communities occurs in line with the appearance of hub regions in the order of bilateral entorhinal cortices, parahippocampal and fusiform gyri, and lateral temporal regions.

**Conclusion:**

Longitudinal tau propagation depicts distinct spreading pathways of the early- and late-onset AD spectrum characterized by the specific location and appearance period of several hub regions that dominantly provide tau.

**Supplementary Information:**

The online version contains supplementary material available at 10.1186/s13195-022-01061-0.

## Introduction

Although an age-related increase in the incidence and prevalence of clinically diagnosed Alzheimer’s disease (AD) has been noted [1–3], up to 5% of AD patients develop symptoms before the age of 65 years. Such patients are considered to have a distinct AD subtype called early-onset AD (EOAD) [4]. Compared to the late-onset AD (LOAD), which predominantly presents with memory dysfunction, EOAD patients are more likely to show greater impairment in non-memory functions such as language, visuospatial, executive, and attention functions [5–10], all of which deteriorate more rapidly [7].

Although the underlying biological mechanisms for these distinct clinical characteristics have yet to be confirmed, recent studies have proposed several models explaining such mechanisms. Patients with EOAD show more prominent hypometabolism in the diffuse lateral temporo-parietal, occipital, precuneus, and posterior cingulate cortices [11, 12] and greater cortical atrophy, particularly in the parieto-occipital cortex [5, 13], while LOAD patients tend to suffer from dominant hypometabolism and cortical atrophy in the medial temporal regions. Moreover, compared with members of the LOAD group, EOAD patients in a postmortem study exhibited greater neurofibrillary tangle (NFT) burden in the middle frontal and inferior parietal cortices [14] and greater tau positron emission tomography (PET) tracer uptake in the frontal and parieto-occipital cortex, while showing no clear difference in the regions corresponding to Braak’s NFT stage I–IV [9, 15, 16]. In a longitudinal tau PET study, younger patients exhibited greater increases in tau tracer uptake in the temporal meta-region of interest [17]. Tau burden and its topographic distribution pattern are reportedly closely related to clinical severity and phenotype [18, 19]. Given that cortical hypermetabolism and atrophy are also locally associated with tau distribution and mediate the effects of tau pathology on cognitive deficits [18–25], a distinct pattern of cortical tau pathology can be expected to be a key factor in the clinical and neuroimaging characteristics of EOAD.

Pathological tau protein travels across synapses [26, 27], and tau distribution patterns spatially overlap with large-scale brain network [28, 29]. Functionally interconnected brain regions exhibited similar levels of tau burden in cross-sectional tau PET studies and were correlated with an increase in tau accumulation in a longitudinal study [30–32], and even the future accumulation of tau has been predicted by functional connectivity [32]. These observations suggest that pathological tau proteins may spread throughout large-scale brain networks. However, little is known about the intrinsic networks through which tau spreading occurs during EOAD and LOAD progression. Moreover, a tau distribution pattern predicted by a spreading model based on a predefined brain network exhibits discrepancies when compared with a real network [33, 34]. These discrepancies can be partially explained by the effects of regional amyloid distribution [34], microglial activation [35], or regional vulnerability in genetic factors [33], but intrinsic tau-spreading pathways that reflect those components have yet to be identified.

We hypothesized that distinct tau-propagation networks exist between the EOAD and LOAD spectra, and attempted to identify the data-driven tau-spreading pathways using longitudinal tau PET data. The identified tau-spreading network encodes potential interregional influences between entire brain regions, and successive changes across the estimated disease progression may reveal the spatiotemporal dynamics involved in tau propagation. We therefore aimed to investigate where and when does tau spread through gateways that drive tau propagation, which might be distinct in EOAD and LOAD progression. A multilayer community-detection method was employed to examine such gateways among the spreading networks.

## Materials and methods

### Participants

We enrolled 142 participants who completed baseline and follow-up tau PET examinations at Gangnam Severance Hospital from January 2015 to March 2019. All participants underwent two PET (^18^F-flortaucipir for tau and ^18^F-florbetaben for amyloid-beta (Aβ)) and magnetic resonance imaging (MRI) scans, and neuropsychological tests [36] at both baseline and follow-up. Based on baseline Aβ-positivity as determined by the agreement of two nuclear medicine specialists, validated visual assessments [37, 38], and neuropsychological tests, baseline Aβ-positive cognitively impaired (CI) individuals with amnestic presentation were identified using diagnostic criteria supplied by the National Institute on Aging and Alzheimer’s Association (“mild cognitive impairment due to AD with intermediate or high likelihood” for prodromal AD and “probable AD dementia with evidence of the AD pathophysiological process” for AD dementia) [39, 40]. We referred to symptomatic patients included in the AD spectrum as CI individuals. Cognitively unimpaired (CU) individuals were healthy volunteers who achieved normal cognition on neuropsychological tests and for whom no abnormality was evident in MRI at baseline. According to the age at onset, the CI group was divided into early-onset (EOCI: onset age < 65 years) and late-onset (LOCI: onset age ≥ 65 years) groups. Onset age was determined through an interview with family members or caregivers of each CI individual. Similarly, the CU group was also divided into young (YCU: baseline age < 65) and old (OCU: baseline age ≥ 65 years) groups. Ultimately, 30 YCU, 44 OCU, 15 EOCI, and 53 LOCI individuals were enrolled in this study.

### Acquisition of PET and MRI scans

Images from PET scans were acquired in a Biograph mCT PET/CT scanner (Siemens Medical Solutions, Malvern, PA, USA) for 20 min at 80 min after injection of ^18^F-flortaucipir and 90 min after injection of ^18^F-florbetaben. After correcting for attenuation with computed tomography images, three-dimensional (3D) PET images were reconstructed using the ordered-subsets expectation maximization algorithm in a 256 × 256 × 223 matrix with 1.591 × 1.591 × 1 mm voxel size. A 3.0 Tesla MRI scanner (Discovery MR750; GE Medical Systems, Milwaukee, WI, USA) was used to produce axial T1-weighted brain scans with 3D-spoiled gradient-recalled sequences (512 × 512 matrix with voxel spacing of 0.43 × 0.43 × 1 mm).

### Image processing steps

Using FreeSurfer 5.3 software (Massachusetts General Hospital, Harvard Medical School; http://surfer.nmr.mgh.harvard.edu), participant-specific volumes-of-interest (VOIs) were created with T1-weighted MRI scans as described in our previous study [41]. In brief, MRI scans were resliced to FreeSurfer space (a 256 × 256 × 256 matrix with 1 mm isovoxels) and then corrected for inhomogeneity. After segmentation of gray and white matter, 3D surfaces were created with trigons. Finally, participant-specific composite VOIs were created with the cortical areas parcellated using curvature information under the guidance of the Desikan–Killiany atlas [42], and subcortical regions were segmented using probabilistic registration [43].

Statistical parametric mapping 12 (Wellcome Trust Centre for Neuroimaging, London, UK) and in-house software implemented in MATLAB 2017b (MathWorks, Natick, MA, USA) were used for integrative processing of ^18^F-flortaucipir PET images. These images were first co-registered to MRI counterparts in FreeSurfer space, and then corrected for partial volume effect (PVE) using the region-based voxel-wise method [44]. Finally, we created PVE-corrected standardized uptake value ratio (SUVR) images with the cerebellar crus median obtained from spatially normalized PET images as a reference, and regional SUVR values for the regions defined by the Desikan–Killiany cortical atlas [42].

Regional ^18^F-flortaucipir SUVR values were then converted to W-scores representing regional tau burdens and compared with controls after adjusting for covariates [45–48]. Multiple linear regression models were created for each region with the regional SUVR values as the outcome and baseline age, sex, and years of education as predictor variables in the Aβ-negative CU group. Residuals were then calculated for each participant accompanied by the individual outcome and predictor variables and divided by the standard deviation (SD) of the residuals obtained from the Aβ-negative CU individuals. The same regression models were applied to calculate W-scores for the follow-up data, and annual changes in W-score (*Δ*W/year) were calculated.

### Construction of tau-spreading networks using pseudo-longitudinal order

**Fig. 1:**
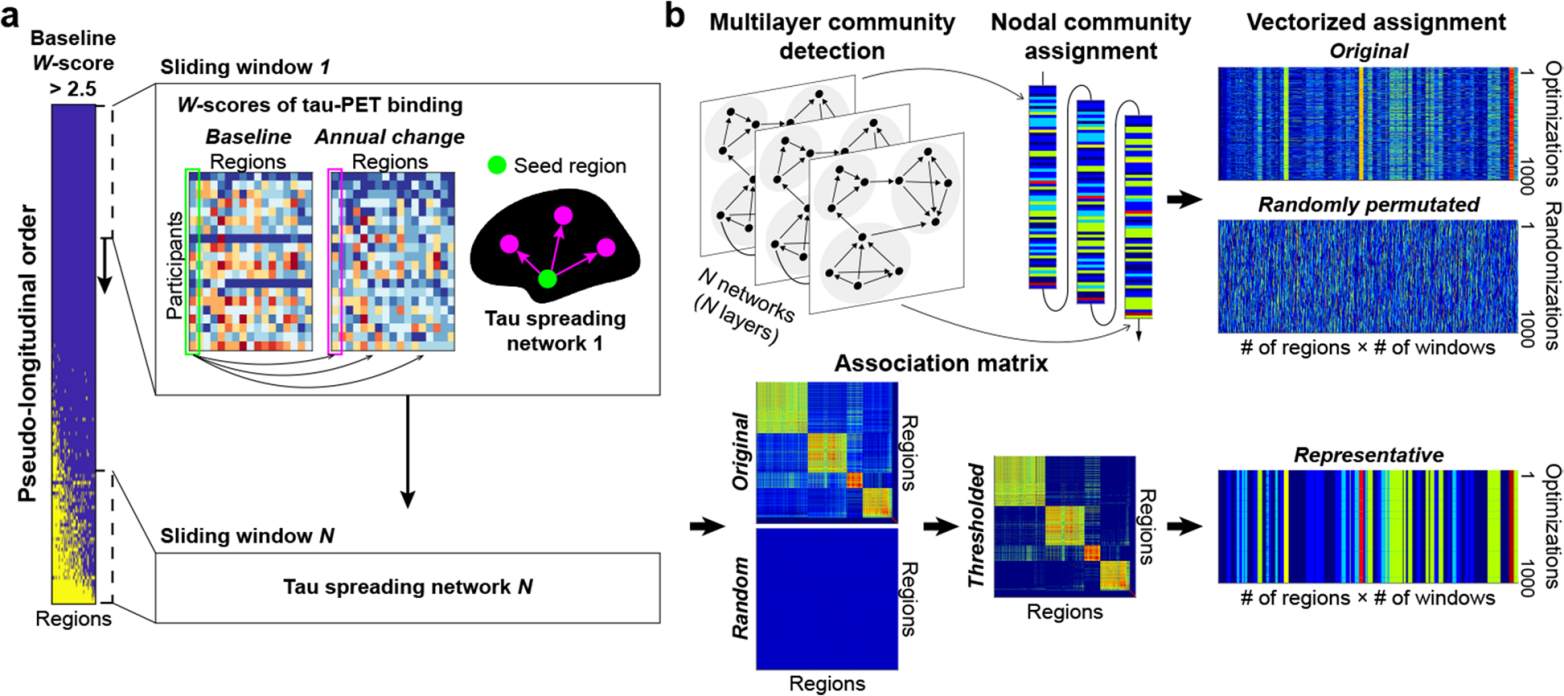
Study overview. **a** All individuals in each age group were sorted by the extent of baseline tau burden to present a pseudo-longitudinal order of disease progression. For all subjects involved in each window moving across the pseudo-longitudinal order, DTGR approach was applied between baseline W-scores of a seed region and annual changes in W-score of another region to construct a tau spreading network. **b** We optimized community structures by maximizing a modularity function, considering internetwork connections between the consecutive tau spreading networks. Due to the heuristic nature of the optimization algorithm, the optimization process was repeated 1000 times and every single node of each network was assigned a community at each iteration. A regional association matrix was then constructed from 1000 original assignments and 1000 randomly permuted assignments, respectively. The original association matrix was thresholded by a maximum value of the random association matrix, and a representative assignment was determined by applying community detection algorithm to the thresholded association matrix

An illustrative figure of the proposed methods is represented in Fig. 1. All participants were sorted in an ascending order by the number of regions with a baseline tau-PET W-score greater than 2.5 [41, 49], creating a pseudo-longitudinal order of disease progression. Participants with the same number of supra-threshold regions were sorted additionally by the median value of W-scores across all regions. To investigate stage-dependent tau propagation networks, we selected a subgroup of subjects using the sliding window method, in which we moved a window for a fixed number of ordered subjects. We applied the sliding window to two age groups: YCU/EOCI (*n* = 45) and OCU/LOCI (*n* = 97). The sliding window was designed to include 40 subjects and moved by one subject from the left (earlier in the pseudo-longitudinal order) to the right (later in the pseudo-longitudinal order). In the case of YCU/EOCI, the window was designed to include 20 subjects due to the smaller number of subjects in the group.

Directional graph theory regression (DTGR) was applied to all subjects in the pseudo-longitudinal order. A longitudinal model of DTGR was used to infer inherent tau spreading networks between two regions taking into account network temporal directionality [50]. For each sliding window, we calculated Spearman correlation coefficients between the baseline W-scores in a seed region (*i*) and the annual W-score change rates in another region (*j*). Because those two regional values were ordered temporally with each other, the correlation coefficient worked as a weight of an edge from region *i* to *j*. We called this directed network a tau-spreading network for each sliding window. For the tau-spreading networks of all sliding windows, we selected the edges with a *P*-value < 0.005, but a range of different *P*-value thresholds were also tested to evaluate reliability. We considered only positive coefficients implying that current accumulation of tau in one region may affect future accumulation in the connected region, which is referred to as tau spreading. Furthermore, the connecting edges were excluded if the target region has a negative mean annual change indicating a decrease in tau over time. The sliding windows were divided into five segments, and mean tau-spreading networks for each segment were constructed using only consistent edges that more than half the windows have within the segment to prevent analytical disturbances from unstable edges along disease progression.

### Multilayer community detection

Using the resulting tau-spreading networks, the brain regions were grouped into sets such that regions were more densely connected to each other than by chance (Fig. 1b). A modular structure (community) was identified based on modularity maximization [51]. To consider temporal dependency across the sliding windows, we defined a multilayer modularity for the directed graph [52, 53]:

$$\boldsymbol{Q}=\frac{\mathbf{1}}{\mathbf{2}\boldsymbol{\mu }}\sum_{\boldsymbol{ij}\boldsymbol{s}\mathbf{r}} \nolimits \left\{\left({\boldsymbol{A}}_{\boldsymbol{ij}\boldsymbol{s}}-{\boldsymbol{\gamma}}_{\boldsymbol{s}}{\boldsymbol{P}}_{\boldsymbol{ij}\boldsymbol{s}}\right){\boldsymbol{\delta}}_{\boldsymbol{s}\boldsymbol{r}}+{\boldsymbol{\delta}}_{\boldsymbol{ij}}{\boldsymbol{C}}_{\boldsymbol{jsr}}\right\}\boldsymbol{\delta} \left({\boldsymbol{g}}_{\boldsymbol{is}},{\boldsymbol{g}}_{\boldsymbol{jr}}\right),$$

where *μ* is the total edge weight in the network, *A*_*ijs*_ is the adjacency between node *i* and *j* in layer *s*, *γ*_*s*_ is the weight of intralayer connections (structural resolution parameter), *P*_*ijs*_ is the component of the corresponding null model matrix, *δ*_*ij*_ is the Kronecker delta symbol, *C*_*jsr*_ is the connection strength between node *j* in slice *s* and slice *r* (interlayer coupling parameter), and *g*_*is*_ is the community assignment of node *i* in layer *s*. We set parameters *γ* and *ω* to 1, which is a frequently used default value [54–56].

A Louvain-like greedy community-detection algorithm [52, 57, 58] was used to determine the optimal modularity function, Q. This optimizing method was iterated until the resulting community structure did not change from one iteration to the next, and a post-processor function was applied to ensure convergence [59]. Due to the heuristic nature of the algorithm, we repeated the optimization process 1000 times for each group. We then constructed a representative community structure based on a comparison with null models to deal with the degeneracy [60]. We first constructed a regional association matrix (a frequency matrix in which any two regions are assigned to the same community across the repetitions) from original assignments and randomly permuted assignments. We obtained a thresholded regional association matrix by subtracting the maximum value of the random association matrix from the original association matrix. A representative assignment was determined by conducting a Louvain-like algorithm with the thresholded association matrix.

Each community was then characterized by its hub regions, which were expected to lead overall spreading of tau pathology within the community. We performed a seed-based analysis for a community of each tau-spreading network. Assuming that a higher number of paths departing from a seed region indicates a greater ability to provide pathology to the connected regions, the regional out-degree was calculated as the number of edges that originated from the seed region. We considered a region with a relatively higher out-degree (> mean + 1.5 SD of out-degrees from all intracommunity seed regions) as a tau-providing hub. Because a region with only a few influential connections can be identified as a hub due to a low number of pathways overall within the community, we excluded regions with fewer than three connections or those appearing as hubs only in a single window.

### Statistical analysis

We used MATLAB 2019a for statistical analysis of demographic data. For between-group comparisons, a Wilcoxon rank sum test was used for continuous variables, and chi-square tests were used for categorical variables.

## Results

### Demographic characteristics

Although the OC group exhibited slightly lower mini-mental state examination (MMSE) scores compared with the YC group (*p* = 0.0036), no differences in sex ratio, years of education, clinical dementia rating sum-of-boxes (CDR-SB), frequencies of the ApoE ε4 genotype, or follow-up intervals between the older groups (OCU or LOCI) and their corresponding younger groups (YCU or EOCI) were evident. The ranges of cognitive decline (MMSE and CDR-SB) in both CI groups are detailed in Fig. S1. Sex and years of education did not differ between the CI and corresponding CU groups, but the CI groups were older (younger group: *p* = 0.0052, older group: *p* = 5e−4) and had worse MMSE (younger group: *p* = 6e−6, older group: *p* = 3e−10) and CDR-SB (younger group: *p* = 1e−10, older group: *p* = 7e−19) scores, higher frequencies of the ApoE ε4 genotype (younger group: *p* [statistics] = 8e−4 [11.250], older group: *p* [statistics] = 0.0015 [10.067]), and shorter follow-up intervals (younger group: *p* = 0.0038, older group: *p* = 6e−5) compared with their corresponding CU groups. Detailed demographic characteristics are provided in Table 1.

**Table 1:** Demographic and clinical characteristics of the study population

Variable	YCU	OCU	EOCI	LOCI
***n***	30	44	15	53
**Age (years)**	58.3 ± 5.5	72.8 ± 6.2^b^	62.8 ± 6.0^a^	76.4 ± 5.3^ab^
**Age at onset (years)**	n. a.	n. a.	59.4 ± 5.6	73.6 ± 5.3^b^
**Females,*n*(%)**	19 (63.3)	26 (59.1)	10 (66.7)	33 (62.3)
**Education (years)**	13.3 ± 3.9	11.6 ± 4.7	11.6 ± 5.0	10.5 ± 5.1
**MMSE**	28.9 ± 1.5	27.8 ± 1.8^b^	21.5 ± 6.2^a^	22.8 ± 4.2^a^
**CDR-SB**	0.0 ± 0.0	0.0 ± 0.0	3.3 ± 2.3^a^	2.9 ± 1.8^a^
***APOE*ε4 carrier,*n*(%)**	5 (16.7)	8 (18.2)	10 (66.7)^a^	26 (49.1)^a^
**Amyloid positivity,*n*(%)**	1 (3.3)	6 (13.6)	15 (100)^a^	53 (100)^a^
**Follow-up interval (months)**	26.0 ± 3.6	25.1 ± 3.6	23.4 ± 1.5^a^	23.0 ± 1.4^a^

Data are presented as mean ± standard deviation. Significant between-group-differences are marked with “^a^” for YCU vs*.* EOCI and OCU vs*.* LOCI, and “^b^” for YCU vs. OCU and EOCI vs*.* LOCI

*Abbreviations*: *YCU/OCU* young and old cognitively unimpaired, *EOCI/LOCI* cognitively impaired due to early- and late-onset Alzheimer’s disease, *MMSE* mini-mental state examination, *CDR-SB* clinical dementia rating sum-of-boxes

### Distinct tau accumulation patterns for AD onset age

**Fig. 2:**
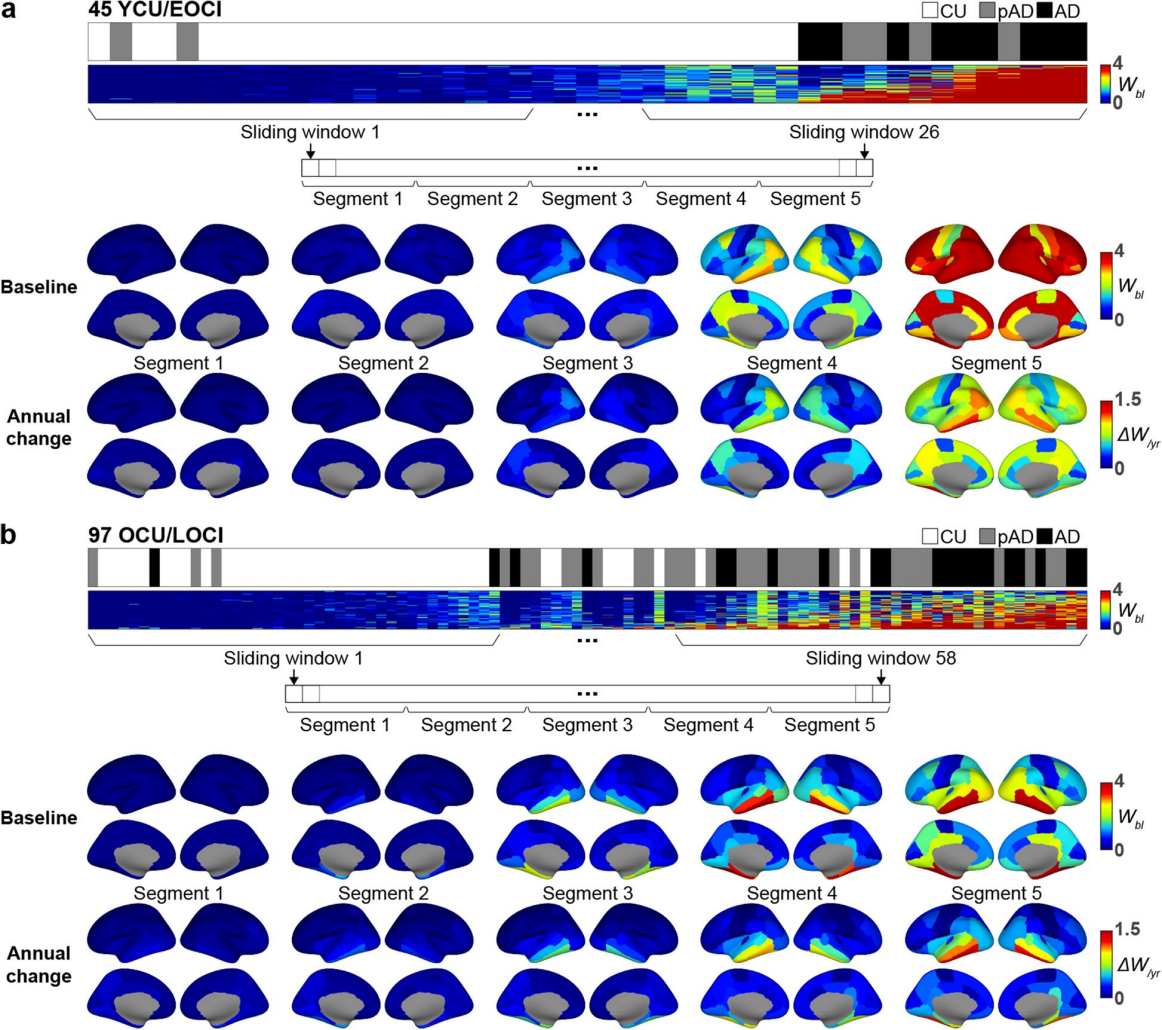
Baseline and annual changes in tau burden in the imaginary spectrum of disease progression in EOAD and LOAD patients. For each YCU/EOCI (**a**) and OCU/LOCI (**b**) group, we divided all individuals across the entire spectrum into five segments. Baseline W-score maps (W_bl_) are displayed in the upper rows and the maps for the annual change in W-score (*Δ*W/year) are in the lower rows. The *y*-axis of the horizontal spectrum bars represents each region, and the *x*-axis represents individual subjects. Abbreviations: YCU, OCU, young and old cognitively unimpaired; EOCI, LOCI, cognitively impaired due to early- and late-onset Alzheimer’s disease; pAD, prodromal Alzheimer’s disease; AD, Alzheimer’s disease dementia

Figure 2 depicts the differential accumulation pattern for tau between the YCU/EOCI and OCU/LOCI groups. When descriptively compared with the baseline W-score and its annual change rate maps within the first segment of the YCU/EOCI group, tau first accumulated in the medial and lateral temporal and the inferior parietal cortex, and extended to the precuneus and posterior cingulate cortices in segment 3 to 4. The annual change rate in the parietal cortex was similar to that of the temporal cortex. Overall, YCU/EOCI group experienced more dramatic accumulation of tau in the diffuse cortical regions (Fig. 2a). However, tau first appeared in the medial temporal lobes of members of the OCU/LOCI group, followed by the inferior temporal and fusiform cortex in segment 2 and 3. It then expanded to the posterior cingulate and inferior parietal cortex in segment 4 and finally reached the remaining cortical regions in segment 5. The maps for the annual change rate in W-scores exhibited patterns similar to those of the baseline maps, but prominent changes were restricted to the temporal regions (Fig. 2b).

### Dynamics of tau spreading through pseudo-longitudinal order

**Fig. 3:**
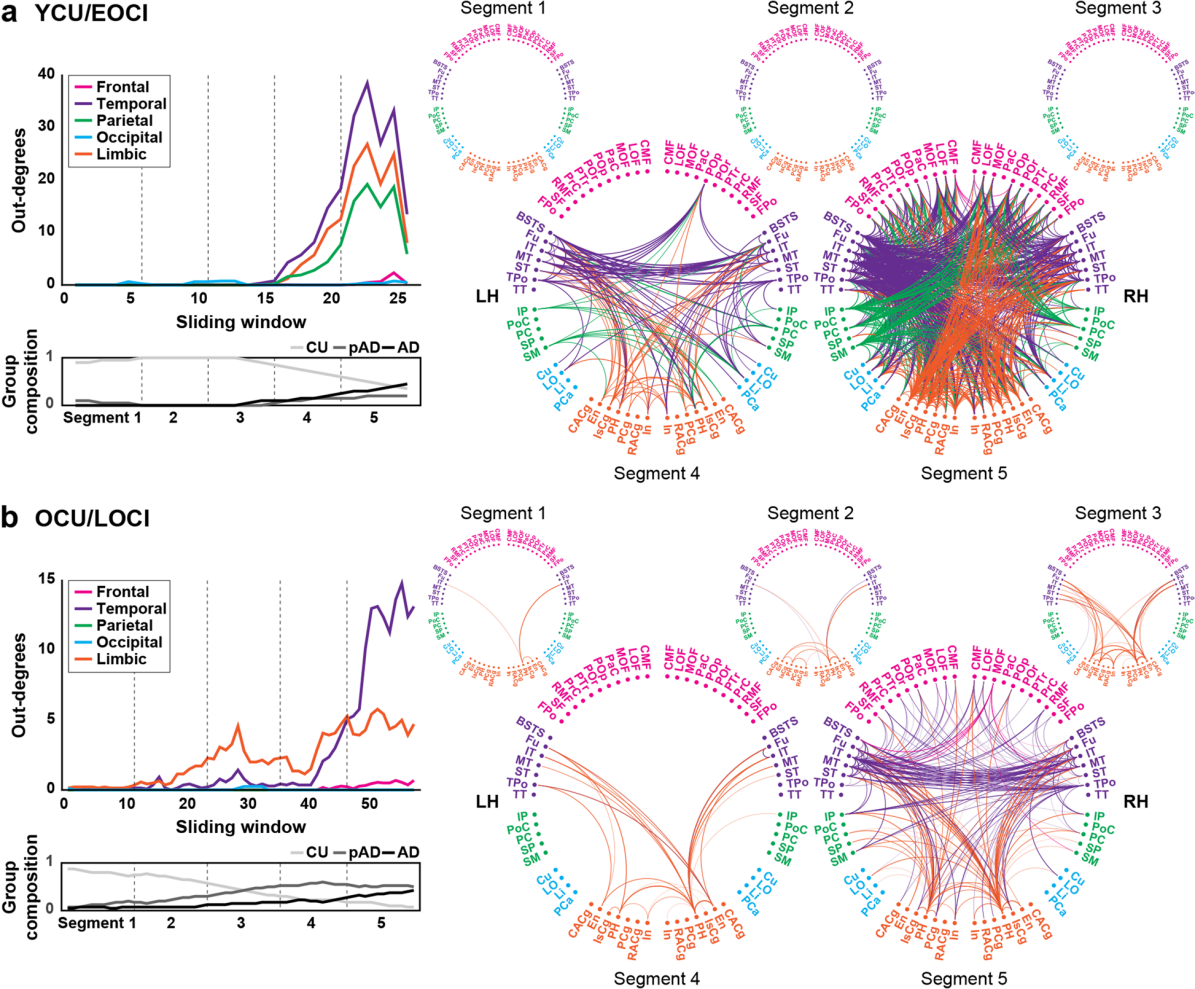
Dynamics of tau spreading across the pseudo-longitudinal order in YCU/EOCI and OCU/LOCI. Left column displays mean number of edges departed from each lobar area and group composition across tau spreading networks for YCU/EOCI (**a**) and OCU/LOCI (**b**) groups. Averaged brain-wide network for each segment is represented using connectogram in right column. Regions are labeled with the abbreviations of the region names in the Desikan-Killiany cortical atlas and colors for their corresponding cortical lobes. Lines connecting two regions (out-edges) are marked with the lobar colors for their origin. Abbreviations: YCU and OCU, young and old cognitively unimpaired; EOCI and LOCI, cognitively impaired due to early- and late-onset Alzheimer’s disease; pAD, prodromal Alzheimer’s disease; AD, Alzheimer’s disease dementia; LH, left hemisphere; RH, right hemisphere. Region labels: frontal (CMF = caudal middle frontal, LOF = lateral orbitofrontal, MOF = medial orbitofrontal, PaC = paracentral, POp = pars opercularis, POr = pars orbitalis, PTr = pars triangularis, PrC = precentral, RMF = rostral middle frontal, SF = superior frontal, FPo = frontal pole), temporal (BSTS = banks of the superior temporal sulcus, Fu = fusiform, IT = inferior temporal, MT = middle temporal, ST = superior temporal, TPo = temporal pole, TT = transverse temporal), parietal (IP = inferior parietal, PoC = postcentral, PC = precuneus, SP = superior parietal, SM = supramarginal), occipital (Cu = cuneus, LO = lateral occipital, Li = lingual, PCa = pericalcarine), and limbic (CACg = caudal anterior cingulate, En = entorhinal, IsCg = isthmus cingulate, PH = parahippocampal, PCg = posterior cingulate, RACg = rostral anterior cingulate, In = insula) lobes

In the identified tau-spreading networks of the YCU/EOCI group, edges originated primarily in the temporal cortex, followed by the limbic and parietal cortices, but were seldom found in the frontal and occipital cortices. These appearances were generally robust across a range of different *P*-value thresholds for edge selection (Fig. S2). In segment 4, tau extended from the temporal cortex to the parahippocampal and insula cortices, and weakly to some regions in the parietal cortex, including the inferior parietal cortex, precuneus, and supramarginal gyrus. Conversely, some of the edges that departed from the entorhinal and isthmus cingulate cortex of the limbic cortex reached the temporal cortex. Several pathways originating in the parietal cortex were also remarkable. In segment 5, tau spreading became much more active between the widespread brain regions, even in connections to the frontal or occipital cortices, while keeping the source regions relatively active in the earlier segments (Fig. 3a).

Scans of the OCU/LOCI group revealed different patterns. Most of the out-edges appeared first in the limbic and temporal cortices and predominated in the temporal cortex in the latter part of the windows, which was similarly reproduced at other edge thresholds (Fig. S2). Tau began to spread from the entorhinal and parahippocampal cortices to the inferior temporal and fusiform cortices. Edges between those regions were most active even in segment 4. In segment 5, tau spreading was widespread, predominantly from the temporal cortex, and from the inferior temporal gyrus in particular. Tau burden in the temporal cortex affected the amount of accumulation in the frontal regions, inferior parietal, supramarginal, and cingulate cortices (Fig. 3b). To rule out the possible influence of the different proportion of CI participants and sliding window size, we reproduced these findings using randomly chosen participants from the OCU/LOCI group with the same number and proportion with those of the YCU/EOCI group (see Fig. S3).

### Community structure in tau-spreading networks

**Fig. 4:**
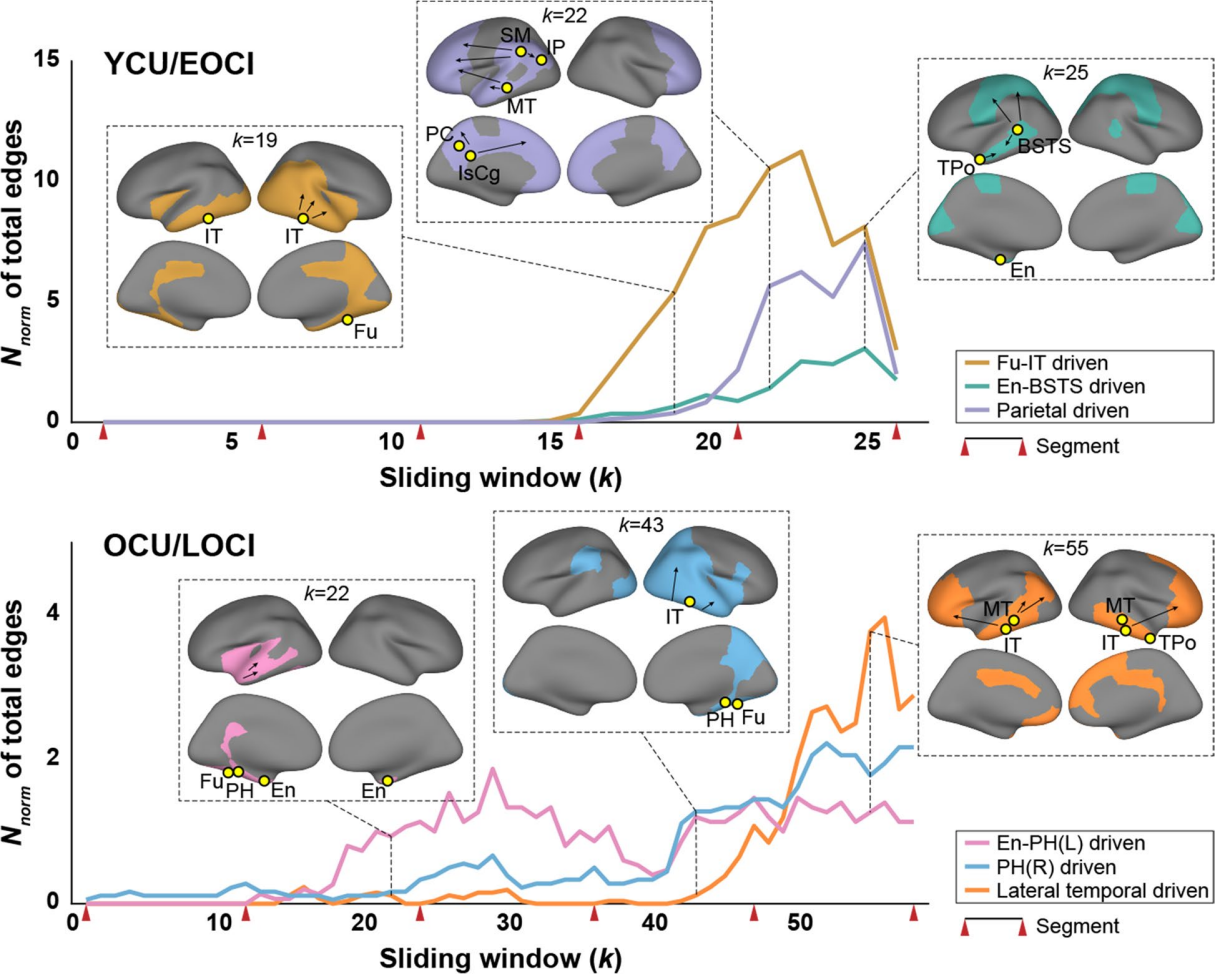
Dynamic community structures in a tau spreading network. Three major communities were found in the latter part of the sliding windows in YCU/EOCI group (upper) and four were found in the OCU/LOCI group (lower), which have distinct changing patterns in the number of overall spreading pathways. For each community, an example map and spreading directions for a specific window are presented in an inset based on the community assignment of that window. The *x*-axes represent each sliding window and the y axes represent total intracommunity edges normalized by the maximum number of regions comprising each community across sliding windows. The yellow circles indicate tau-providing hubs within the corresponding community and the red wedges indicate the boundaries of each segment. Abbreviations: YCU and OCU, young and old cognitively unimpaired; EOCI and LOCI, cognitively impaired due to early- and late-onset Alzheimer’s disease. Abbreviations for the region labels are described in the legend of Fig. 3

**Fig. 5:**
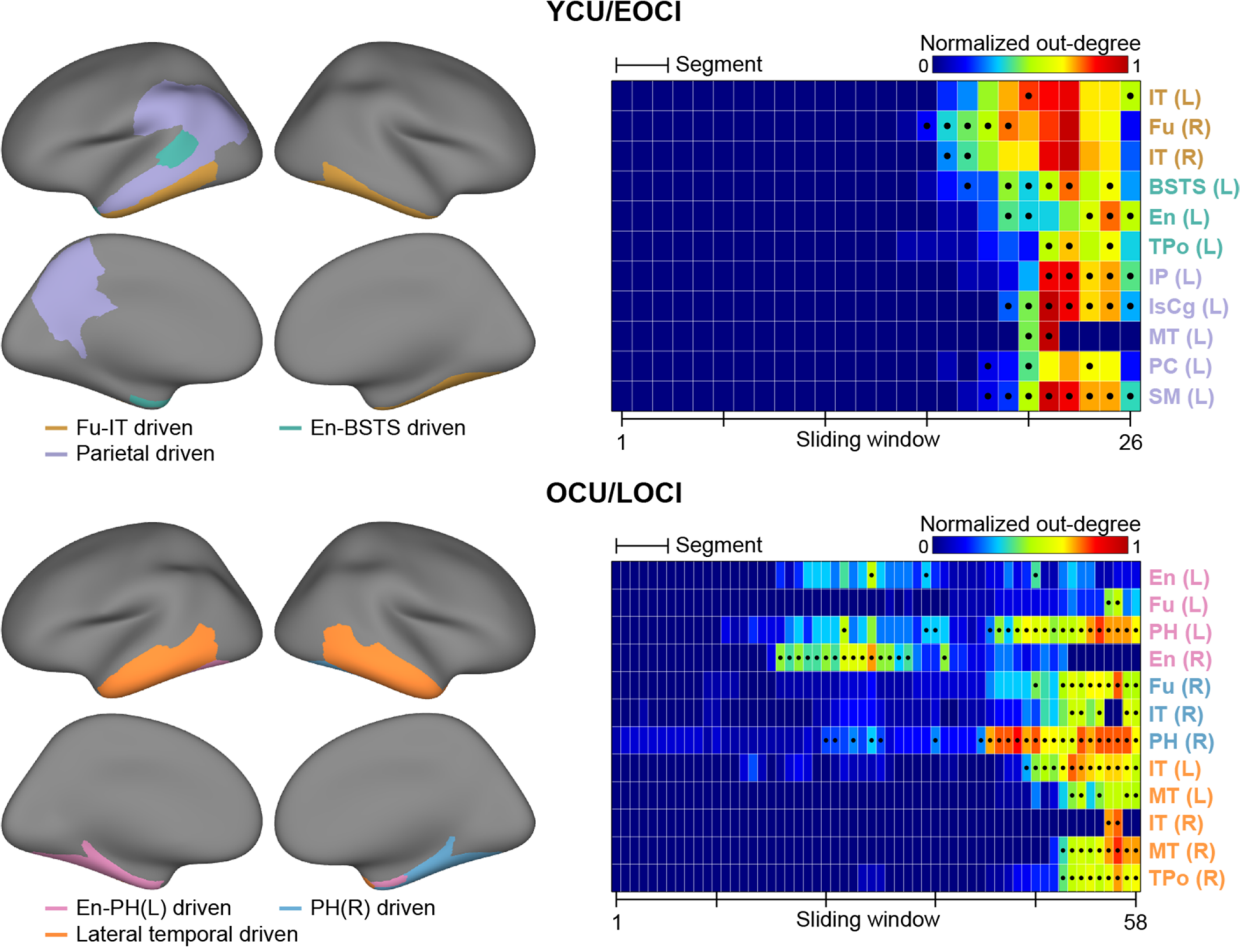
Intracommunity tau-providing hubs determined in each sliding window. The entire tau-providing hubs identified across whole sliding windows were exclusive between the corresponding communities in both YCU/EOCI and OCU/LOCI groups (left). The characteristics of the selected hubs were investigated based on where the hub is defined across the windows and how many regions the hub influences (right). The x axes of the color-coded matrix represent each sliding window and the y axes represent regions selected as hubs. Colors for the matrices represent the out-degrees, normalized by the maximum number of regions comprising each community. The black dots represent the location in which each region was qualified as hub and the black tick marks represent the boundaries of each segment. Abbreviations: YCU and OCU, young and old cognitively unimpaired; EOCI and LOCI, cognitively impaired due to early- and late-onset Alzheimer’s disease. Abbreviations for the region labels are described in the legend of Fig. 3

Across the sliding windows in each group, the tau-spreading networks presented as a converged community structure. The YCU/EOCI group was characterized by three major communities. The regional composition for each community changed slightly across the windows, but presented as a mostly exclusive collection that provided hubs (Figs. 4 and 5). For the first community, the hubs were found mainly in the temporal cortex, including the fusiform and inferior temporal gyri in the windows for segment 4 (“Fu-IT driven”). Left banks of the superior temporal sulcus (BSTS), entorhinal (En), and temporal pole cortices were selected as hubs for the second community (“En-BSTS driven”), and left inferior parietal, isthmus cingulate, middle temporal, precuneus, and supramarginal cortices were identified for the third community (“parietal driven”). As shown in Fig. 4, tau spreading increased first within the Fu-IT driven community along with the earliest emergence of its hubs. The En-BSTS and parietal driven communities followed the Fu-IT driven community in the latter part of the segment 5.

On the other hand, three major communities were identified in the OCU/LOCI group. Left and right entorhinal cortices explained other regions’ tau accumulation changes within the first community in the middle part of segments 2 and 3 (“En-PH(L) driven”) but did not persist in the latter part. By contrast, left/right parahippocampal (PH) cortices, which were identified for the first and second communities, remained active in the middle to last windows (“En-PH[L]/PH[R] driven”). In the latter windows, providing hubs were found for the third community, including both the inferior and middle temporal and right temporal pole cortices (“lateral temporal driven”). Each community in the OCU/LOCI group appeared at different periods, while three major communities of the YCU/EOCI group exhibited nearly identical rising and fading patterns throughout the course of disease severity. These findings were broadly replicated in a subset of participants for the OCU/LOCI group adjusted for the proportion of CI participants and sliding window size in the YCU/EOCI group (see Fig. S3).

## Discussion

We found distinct tau-spreading pathways in EOCI and LOCI due to AD based on the temporal directionality of baseline tau burden and longitudinal tau accumulation rate. Consecutive tau-spreading networks calculated through the pseudo-longitudinal order revealed temporal changes in tau-spreading patterns for the YCU/EOCI and OCU/LOCI groups. Each group-specific multilayer community structure clearly showed how those spreading patterns differed. Both community structures consisted of some communities with unique out-degree hubs, with distinct locations, affecting regions, and active periods between the YCU/EOCI and OCU/LOCI groups. These findings can partly explain the distinct patterns of tau spreading in the EOCI and LOCI groups.

Our results agree with those of previous studies. Across the pseudo-longitudinal order within the OCU/LOCI group, tau pathology first appeared in the medial temporal cortex, the entorhinal cortex in particular, and then the lateral temporal and cingulate cortices, followed by the parietal and frontal areas, showing Braak-like progression [16, 61]. Meanwhile, the YCU/EOCI group exhibited the first appearance of tau accumulation in the diffuse medial and lateral temporal cortices and inferior parietal cortex, along with a higher tau burden and accumulation rate in the diffuse cortical areas in the late stage. Prior studies have reported a higher burden of tau pathology in younger patients [9, 14, 15, 17]. This reconfirms the differences in tau pathology patterns and suggests different tau-spreading processes.

Sequential networks derived by DTGR can reveal systematic tau propagation processes across the spectrum of disease severity. The most remarkable finding from our examination of the YCU/EOCI group involved the out-edges from the parietal region and cingulate cortex, which were largely absent in the OCU/LOCI group, while the edges extending from the medial and lateral temporal cortices were common, although there was a difference in level. In addition to previous reports that the parieto-occipital area or posterior cingulate cortex showed greater tau burden or more-severe atrophying among early-onset dementia patients [5, 9, 15, 62], regional tau uptake appeared to significantly affect the longitudinal pathology accumulation of other brain regions broadly.

In the case of the OCU/LOCI group, the entorhinal cortex and parahippocampal gyrus appeared to begin providing tau to the nearby area at the early period of the spectrum. Within the latter part of the pseudo-longitudinal order corresponding to segment 5, pathways from the temporal lobe emerged markedly and reached the remaining frontal, parietal, and limbic regions. The influences of the entorhinal or parahippocampal regions at relatively earlier periods are consistent with a previous report that pathologic retention of NFT often appears in medial temporal lobe before the onset of symptoms [63]. Meanwhile, the lateral temporal area may play a crucial role in spreading tau among CI individuals.

The community structures exhibited two noticeable differences between the YCU/EOCI and OCU/LOCI groups. First, the community hubs were identified in more diffuse cortical regions, including the temporal, limbic, and parietal lobes in the YCU/EOCI group, while the hubs were confined to the medial and temporal cortices in the OCU/LOCI group. The parietal area, including the precuneus, inferior parietal, and isthmus cingulate cortices, is in the posterior part of the default mode network (DMN), as defined by resting-state functional MRI [64]. An age-related shift of DMN activity from its posterior to anterior part is evident, and younger adults have more preserved posterior DMN activity compared with the elderly [65]. Relatively preserved posterior DMN may therefore induce faster propagation of tau pathology in related regions and may partly explain the high tau burden and tau-providing power of hub regions in the EOCI group.

In transgenic mice expressing pathological tau protein, activated microglia precede the formation `of tau tangles and increased with tau pathology. Conversely, treatment with an immunosuppressant or direct depletion of microglia attenuates tau pathology and tau propagation [66–68]. A post-mortem study exhibited an increase in the tangle burden and number of microglia and astroglia across the entire disease course after the plateau of amyloid plaque load [69]. A ^11^C-PBR28 PET study targeting the translocator protein 18kDa found that EOAD patients were associated with greater binding in the diffuse association cortices, particularly in the prefrontal, inferior parietal, precuneus, and occipital cortices, when compared with LOAD patients [70]. These regions largely overlapped with the identified tau-providing hubs of the YCU/EOCI group in our study. Enhanced neuroinflammation may therefore offer an alternative explanation for high tau burden in the EOCI, and there may be a synergetic effect between the relatively preserved posterior DMN and enhanced neuroinflammation within the hub regions.

The second distinct difference is that in the YCU/EOCI group, three communities seemed to be sustained at the similar periods, while three major communities of the OCU/LOCI group showed distinct rising and fading patterns. Three major communities coexisted independently in the latter disease stages of the YCU/EOCI group, whose tau-providing hub regions maintained high intracommunity out-degrees. Hubs of the Fu-IT driven community were not identified in the latter part due to the overall increasing out-degrees across intracommunity regions, but they retained the highest out-degrees (top 5 within the Fu-IT driven community). Given the widespread location of community hubs in the YCU/EOCI group, the coexistence of the three communities show multiple local distribution centers with their own territories. On the other hand, the hub regions for tau propagation appeared to move sequentially from the entorhinal cortex to the lateral temporal cortices in the case of OCU/LOCI. Although the PH driven and lateral temporal driven communities coexisted in the latter periods, their territories are rather limited on the lateral temporal regions leading a marked edge increases within the last segment of the OCU/LOCI group. Spatially confined hub regions and alternate rising of major communities may imply a relatively limited propagation ability compared with the EOCI group.

## Limitation

With respect to study limitations, the number of subjects, particularly for the EOCI group, was small for a study with this level of complexity. Moreover, given the very small number of preclinical group, our findings may not apply to the whole disease spectrum or cover it only sparsely. However, even when analyzing in a priori defined subgroups stratified by age and AD status, an alternative approach to define the spectrum, both YCU/EOCI and OCU/LOCI groups exhibited markedly different tau-spreading characteristics, including a distinct set of critical hub regions, and may be worth investigating (Fig. S4). Due to imbalance in sample size and the proportion of diagnostic groups between YCU/EOCI and OCU/LOCI groups, those differences, especially at the earliest stages, need to be interpreted with careful consideration. But, even in the case that we matched the sample size and the proportion of diagnostic subgroups by downsampling participants in the OCU/LOCI group, the main findings were largely replicated (Fig. S3). Another limitation arises from our use of only two consecutive PET scans for each subject. Only a few previous studies drew on up to three PET scans. However, the proposed method in the current study can be easily extended to more than two PET scans. Extension of the analysis to more PET scans and a greater dataset may be helpful in future studies.

## Conclusions

Our community-based dynamic network model systematically elucidates the distinct tau-spreading natures of the EOCI and LOCI groups. This data-driven approach suggests that different dominant communities and specific continuation periods drive the distinct tau-spreading processes, distinct tau pathology patterns, and possibly peculiar clinical features of the patient groups showing those distinguishable patterns. Future studies should investigate which underlying factors are related to which differences, including genetic factors, neuronal connectivity, and aging.

## Supplementary Information

**Additional file 1: Figure S1.** Comparison of the ranges of cognitive decline. **Figure S2.** Lobar out-degrees plotted against a range of *p*-value cutoffs for edge selection. **Figure S3.** Replication of tau spreading dynamics and intracommunity tau-providing hubs. **Figure S4.** Tau spreading network and community structure for each diagnostic group.

## Data Availability

The datasets used and analyzed during the current study are available from the corresponding author on reasonable request.

## Abbreviations

AD: Alzheimer’s disease
EOAD: Early-onset Alzheimer’s disease
LOAD: Late-onset Alzheimer’s disease
NFT: Neurofibrillary tangle
PET: Positron emission tomography
Aβ: Amyloid-beta
MRI: Magnetic resonance imaging
CI: Cognitively impaired
CU: Cognitively unimpaired
EOCI: Early-onset cognitively impaired
LOCI: Late-onset cognitively impaired
YCU: Young cognitively unimpaired
OCU: Old cognitively unimpaired
3D: Three-dimensional
VOIs: Volumes-of-interest
PVE: Partial volume effect
SUVR: Standardized uptake value ratio
SD: Standard deviation
DTGR: Directional graph theory regression
MMSE: Mini-mental state examination
CRD-SB: Clinical dementia rating sum-of-boxes
BSTS: Banks of the superior temporal sulcus
En: Entorhinal
PH: Parahippocampal
L: Left
R: Right
DMN: Default mode network
