# Supplementary material for: Dynamic network model reveals distinct tau spreading patterns in early- and late-onset Alzheimer disease
Source: Alzheimers Res Ther. 2022 Sep 2;14:121. doi: 10.1186/s13195-022-01061-0 (PMC9438183; doi:10.1186/s13195-022-01061-0)
Supplement: Supplementary file 1 — Additional file 1: Figure S1. Comparison of the ranges of cognitive decline. Figure S2. Lobar out-degrees plotted against a range of p-value cutoffs for edge selection. Figure S3. Replication of tau spreading dynamics and intracommunity tau-providing hubs. Figure S4. Tau spreading network and community structure for each diagnostic group. [file 13195_2022_1061_MOESM1_ESM.docx]

**Figures**


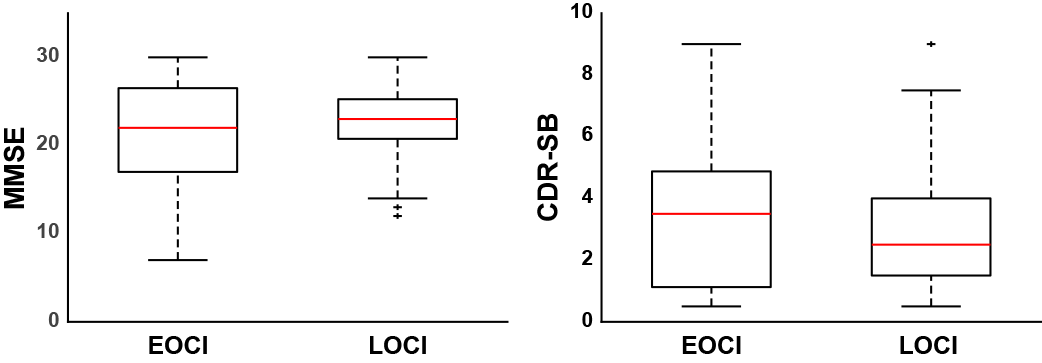


**Figure S1. Comparison of the ranges of cognitive decline.** Box plots represent the ranges of MMSE and CDR-SB scores for each EOCI and LOCI group. Abbreviations: MMSE, mini-mental state examination; CDR-SB, clinical dementia rating sum-of-boxes; EOCI, LOCI, cognitively impaired due to early- and late-onset Alzheimer’s disease


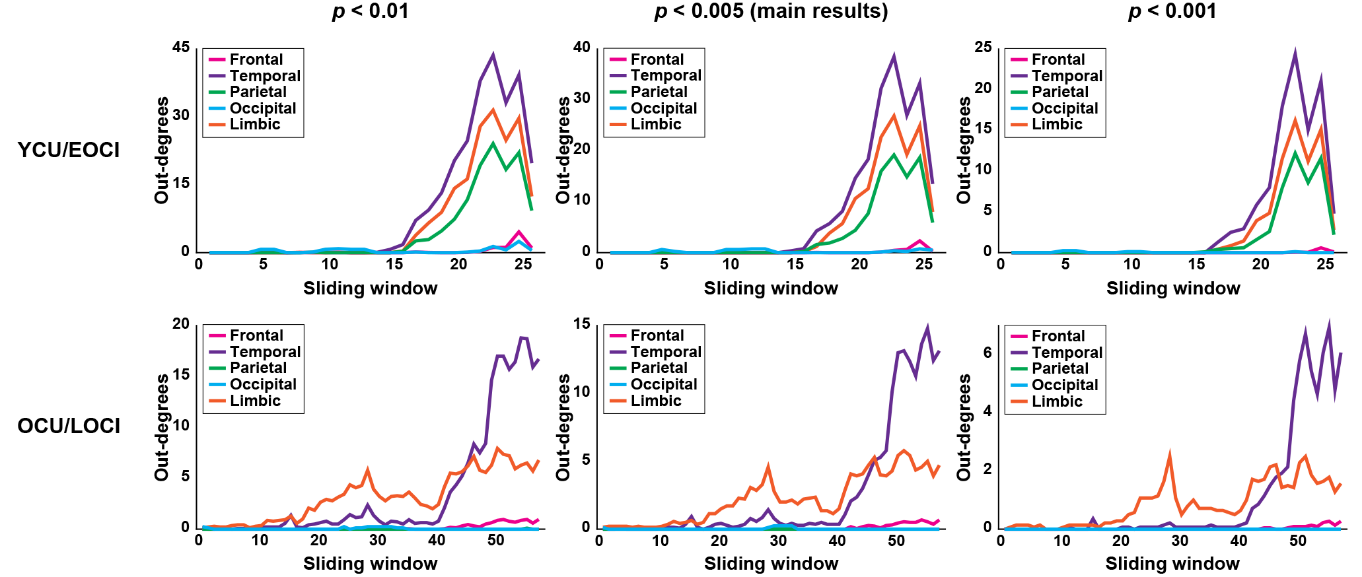


**Figure S2. Lobar out-degrees plotted against a range of p-value cutoffs for edge selection.** Out-degrees were averaged across each lobar regions in tau spreading networks using three different *P* value cutoffs (0.01, 0.005, and 0.001) for selection of edges. Abbreviations: YCU, OCU, young and old cognitively unimpaired; EOCI, LOCI, cognitively impaired due to early- and late-onset Alzheimer’s disease

**
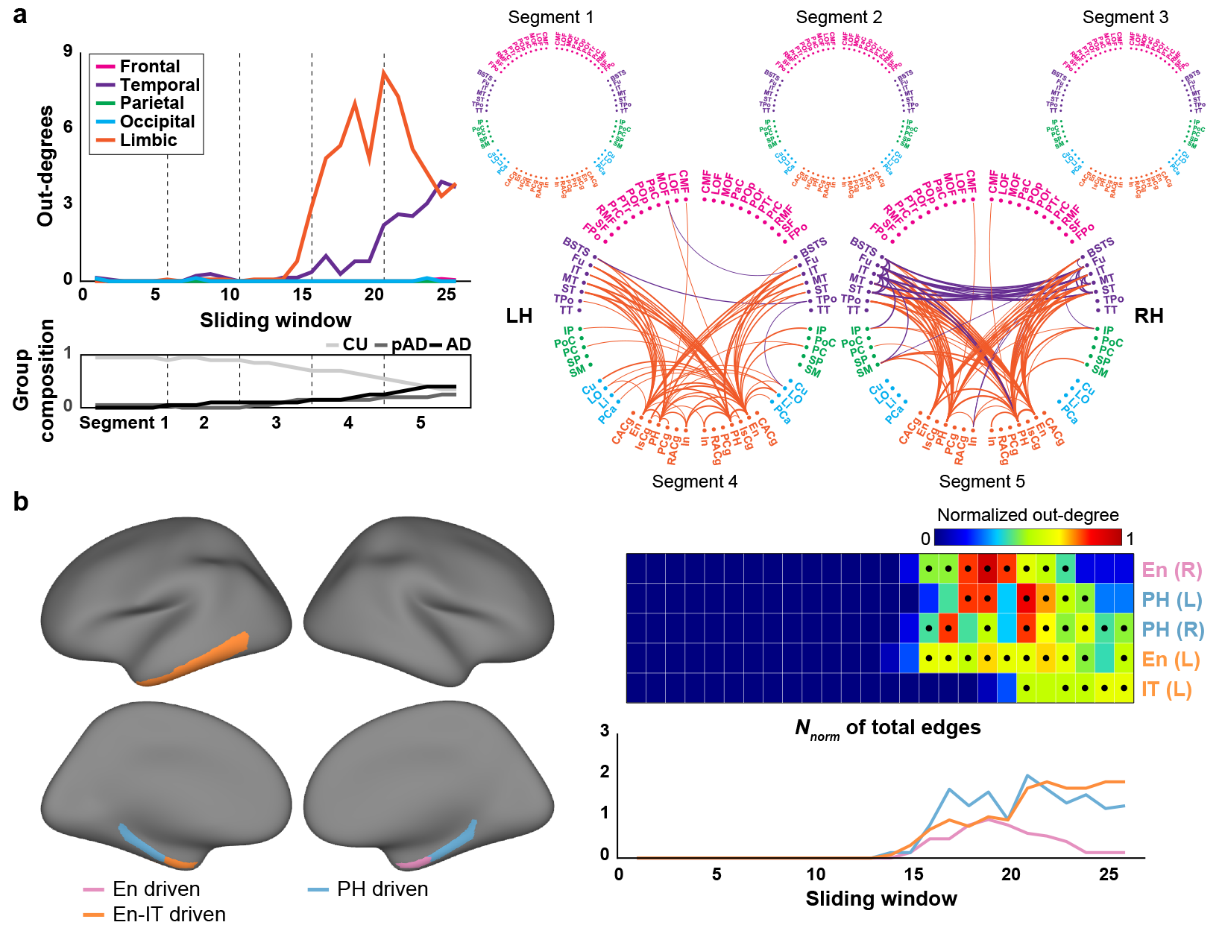
**

**Figure S3. Replication of tau spreading dynamics and intracommunity tau-providing hubs. a.** Key findings in OCU/LOCI group were reproduced with diagnostic group proportions and a window size matched with those of the YCU/EOCI group. Mean lobar out-degree graph with group composition information and tau spreading network connectogram for each segment mirror the format of **Fig. 3**. **b.** Characteristics of the identified tau-providing hubs were represented in a similar format of **Figs. 4 and 5**. Abbreviations: CU, cognitively unimpaired; pAD, prodromal Alzheimer’s disease; AD, Alzheimer’s disease dementia; LH, left hemisphere; RH, right hemisphere. Abbreviations for the region labels are described in the legend of **Fig. 3**

**
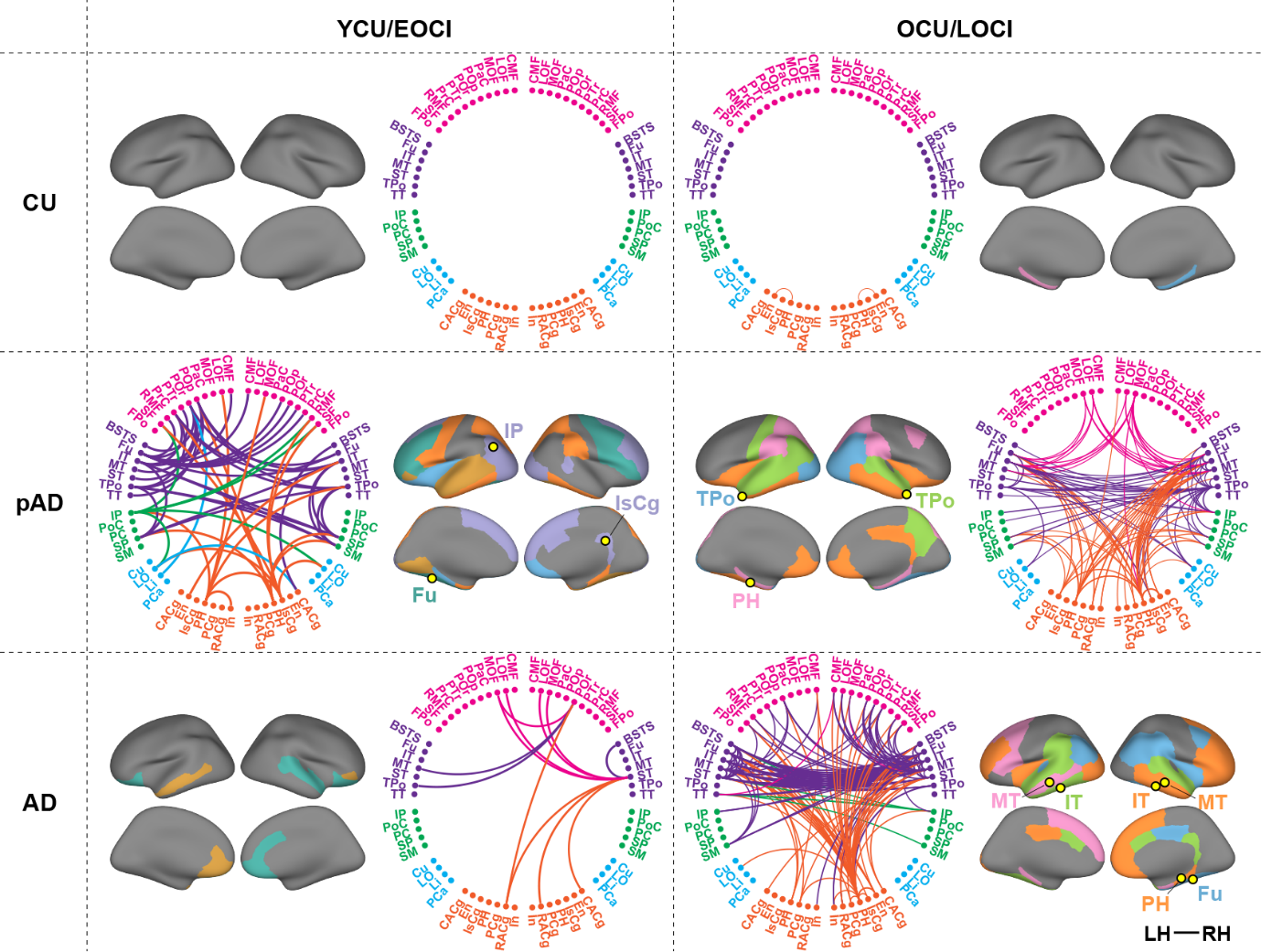
**

**Figure S4. Tau spreading network and community structure for each diagnostic group.** For each disease group, a brain-wide network is represented using connectogram and the corresponding tau-providing hubs were identified. Abbreviations: YCU, OCU, young and old cognitively unimpaired; EOCI, LOCI, cognitively impaired due to early- and late-onset Alzheimer’s disease; pAD, prodromal Alzheimer’s disease; AD, Alzheimer’s disease dementia; LH, left hemisphere; RH, right hemisphere. Abbreviations for the region labels are described in the legend of **Fig. 3**
